# Supplementary material for: Resolving the full spectrum of human genome variation using Linked-Reads
Source: Genome Res. 2019 Apr;29(4):635–45. doi: 10.1101/gr.234443.118 (PMC6442396; doi:10.1101/gr.234443.118)
Supplement: Supplemental Material [file supp_gr.234443.118_Supplemental_longranger-2.2.zip › longranger-2.2.tar.bz2!/lr2.2-public/lib/stan/cmdstan/stan/doc/api/html/bfgs__update_8hpp_source.html]

Stan: src/stan/optimization/bfgs\_update.hpp Source File


|  |  |
| --- | --- |
|  | Stan  2.5.0  probability, sampling & optimization |


- Main Page
- Related Pages
- Namespaces
- Classes
- Files

- File List
- File Members

All Classes Namespaces Files Functions Variables Typedefs Enumerations Enumerator Friends Macros Pages

- src
- stan
- optimization

bfgs\_update.hpp

Go to the documentation of this file.

1 #ifndef STAN\_\_OPTIMIZATION\_\_BFGS\_UPDATE\_HPP

2 #define STAN\_\_OPTIMIZATION\_\_BFGS\_UPDATE\_HPP

3

4 #include <stan/math/matrix/Eigen.hpp>

5

6 namespace stan {

7  namespace optimization {

8  template<typename Scalar = double,

9  int DimAtCompile = Eigen::Dynamic>

10  class BFGSUpdate\_HInv {

11  public:

12  typedef Eigen::Matrix<Scalar,DimAtCompile,1> VectorT;

13  typedef Eigen::Matrix<Scalar,DimAtCompile,DimAtCompile> HessianT;

14

26  inline Scalar update(const VectorT &yk, const VectorT &sk,

27  bool reset = false) {

28  Scalar rhok, skyk, B0fact;

29  HessianT Hupd;

30

31  skyk = yk.dot(sk);

32  rhok = 1.0/skyk;

33

34  Hupd.noalias() = HessianT::Identity(yk.size(),yk.size())

35  - rhok\*sk\*yk.transpose();

36  if (reset) {

37  B0fact = yk.squaredNorm()/skyk;

38  \_Hk.noalias() = ((1.0/B0fact)\*Hupd)\*Hupd.transpose();

39  }

40  else {

41  B0fact = 1.0;

42  \_Hk = Hupd\*\_Hk\*Hupd.transpose();

43  }

44  \_Hk.noalias() += rhok\*sk\*sk.transpose();

45  return B0fact;

46  }

47

56  inline void search\_direction(VectorT &pk, const VectorT &gk) const {

57  pk.noalias() = -(\_Hk\*gk);

58  }

59

60  private:

61  HessianT \_Hk;

62  };

63  }

64 }

65

66 #endif

stan::optimization::BFGSUpdate\_HInv::HessianT

Eigen::Matrix< Scalar, DimAtCompile, DimAtCompile > HessianT

**Definition:** bfgs\_update.hpp:13

stan::optimization::BFGSUpdate\_HInv::search\_direction

void search\_direction(VectorT &pk, const VectorT &gk) const

Compute the search direction based on the current (inverse) Hessian approximation and given gradient...

**Definition:** bfgs\_update.hpp:56

stan::optimization::BFGSUpdate\_HInv

**Definition:** bfgs\_update.hpp:10

Eigen.hpp

stan::optimization::BFGSUpdate\_HInv::VectorT

Eigen::Matrix< Scalar, DimAtCompile, 1 > VectorT

**Definition:** bfgs\_update.hpp:12

stan::optimization::BFGSUpdate\_HInv::update

Scalar update(const VectorT &yk, const VectorT &sk, bool reset=false)

Update the inverse Hessian approximation.

**Definition:** bfgs\_update.hpp:26


---

[ Stan Home Page ]

*© 2011–2014,
Stan Development Team.*
